# Driving Fatigue Detection with Three Non-Hair-Bearing EEG Channels and Modified Transformer Model

**DOI:** 10.3390/e24121715

**Published:** 2022-11-24

**Authors:** Jie Wang, Yanting Xu, Jinghong Tian, Huayun Li, Weidong Jiao, Yu Sun, Gang Li

**Affiliations:** 1 Key Laboratory of Urban Rail Transit Intelligent Operation and Maintenance Technology & Equipment of Zhejiang Provincial, Zhejiang Normal University, Jinhua 321004, China; 2 College of Mathematics and Computer Science, Zhejiang Normal University, Jinhua 321004, China; 3 College of Engineering, Zhejiang Normal University, Jinhua 321004, China; 4 College of Teacher Education, Zhejiang Normal University, Jinhua 321004, China; 5 Key Laboratory of Intelligent Education Technology and Application, Zhejiang Normal University, Jinhua 321004, China; 6 Key Laboratory for Biomedical Engineering of Ministry of Education of China, Department of Biomedical Engineering, Zhejiang University, Hangzhou 310058, China; 7 College of Mathematical Medicine, Zhejiang Normal University, Jinhua 321004, China

**Keywords:** driving fatigue detection, electroencephalogram (EEG), transformer, GLU-Oneformer, lateralization, non-hair-bearing (NHB)

## Abstract

Driving fatigue is the main cause of traffic accidents, which seriously affects people’s life and property safety. Many researchers have applied electroencephalogram (EEG) signals for driving fatigue detection to reduce negative effects. The main challenges are the practicality and accuracy of the EEG-based driving fatigue detection method when it is applied on the real road. In our previous study, we attempted to improve the practicality of fatigue detection based on the proposed non-hair-bearing (NHB) montage with fewer EEG channels, but the recognition accuracy was only 76.47% with the random forest (RF) model. In order to improve the accuracy with NHB montage, this study proposed an improved transformer architecture for one-dimensional feature vector classification based on introducing the Gated Linear Unit (GLU) in the Attention sub-block and Feed-Forward Networks (FFN) sub-block of a transformer, called GLU-Oneformer. Moreover, we constructed an NHB-EEG-based feature set, including the same EEG features (power ratio, approximate entropy, and mutual information (MI)) in our previous study, and the lateralization features of the power ratio and approximate entropy based on the strategy of brain lateralization. The results indicated that our GLU-Oneformer method significantly improved the recognition performance and achieved an accuracy of 86.97%. Our framework demonstrated that the combination of the NHB montage and the proposed GLU-Oneformer model could well support driving fatigue detection.

## 1. Introduction

Driving fatigue, which is often caused by engaging in prolonged and monotonous driving tasks, is a common experience for most drivers [1]. Driving fatigue would lead to a decrease in vigilance and reaction capability, which can seriously affect the response and decision-making ability of drivers in dangerous situations, resulting in a significant increase in the error rate of driving operations [2,3]. It has been reported that 94% of the main causes of car accidents were caused by human error [4]. Fatigue driving can greatly increase the occurrence of these errors, which pose a serious threat to people’s life and property safety. According to a survey, 7.0% of all traffic accidents and 16.5% of fatal traffic accidents are related to driving fatigue [5]. Therefore, researchers have constantly been exploring detection methods to reduce the negative impacts of driving fatigue.

The electroencephalogram (EEG) signals can directly reflect brain activity during cognitive tasks, known as the gold standard for fatigue detection [6,7]. Consequently, EEG is often used to explore fatigue detection [8,9,10] and depict the participant’s mental states [10,11,12]. In recent years, some strategies and algorithms that improve the practicability and accuracy of EEG-related driving fatigue detection have been studied and concerned by researchers, mainly around the following three points. Firstly, fewer prefrontal EEG channels were used to improve the practicability of driving fatigue detection [13,14]. Wearing the whole brain electrode cap is inconvenient and impractical in most scenes. One feasible solution is to reduce the number of electrodes. Wei et al. introduced a non-hair-bearing (NHB) montage and only used four channels (Fp1, Fp2, F7, F8) to improve the convenience of EEG recording without interference from hair [15]. To some extent, NHB montage can promote the further exploration and development of EEG devices in the driver fatigue monitoring area. Secondly, the improved feature extraction algorithms were used to extract effective EEG features [16,17,18]. Luo et al. proposed an adaptive multi-scale entropy feature extraction algorithm for signal reconstruction and feature extraction, which uses an adaptive scale factor algorithm to determine the scale factor of the entropy [19]. Gao et al. proposed a relative wavelet entropy complex network(RWECN) to characterize the topological structure of the brain networks by combining the wavelet entropy and the statistical measure of RWECN [20]. Thirdly, efficient classification algorithms were used to improve the accuracy of identifying fatigue states [21,22,23]. Wang et al. combined an attention-based multi-scale convolutional neural network(CNN) with a dynamic graph convolutional network, which could automatically extract features from EEG signals and realize end-to-end mode, achieving a recognition accuracy of 95.65% with 24-channel EEG data [24]. Delvigne et al. proposed a new framework that used transformer architecture and combined time and space information to achieve great recognition results on multiple datasets [25]. 

Previous studies have certified that the brain is lateralized. That is, the left and right brain areas are asymmetric in structure and function [26,27]. The lateralization can enable each hemisphere of the brain to deal with different types of stimuli or different aspects of the same stimulus. Thereby the brain can process more information [28]. Moreover, the use of different information processing and behavior control in each hemisphere can increase the cognitive capacity of humans [28], which may facilitate feature extraction and classification in algorithms. Pane et al. reported that there is lateralization in human emotions in that the left brain focuses on happy and relaxed emotions, the right brain focuses more on sad and angry emotions, and the valence emotional lateralization significantly improves the performance of emotion recognition [29]. Many studies demonstrated that people have different lateralization tendencies when engaging in different tasks [30,31]. In addition, Sun et al. found that there was also asymmetry in the brain during mental fatigue tasks, and the functional connectivity pattern of brain function in the frontoparietal region was more inclined to the right side [11]. Therefore, in this study, we attempted to introduce the lateralization strategy to extract features to improve classification accuracy. 

Recently, transformer architecture has been used in EEG-related research and has powerful performance in the classification task, mainly due to its unique multi-head attention mechanism [32]. However, the direct application of this model will increase the time complexity and raise memory allocation problems, and the computational complexity is the quadratic of the sequence length. Researchers have combined some feature extraction algorithms (such as CNN [33], long short-term memory (LSTM) [34], and multi-resolution CNN [35]) with a multi-head attention mechanism to improve classification accuracy and reduce complexity. In this study, we extracted multidimensional EEG features to avoid time and memory consumption problems and improved the transformer architecture to be more applicable to the one-dimensional feature vector.

In this study, we will adopt the proven NHB montage to improve its practicality [36]. Moreover, we innovatively use the brain lateralization feature and significantly enhanced transformer algorithm to further improve the driving fatigue recognition accuracy to support the NHB strategy for practical applications.

## 2. Materials and Methods

### 2.1. Experimental Design

**Figure 1:**
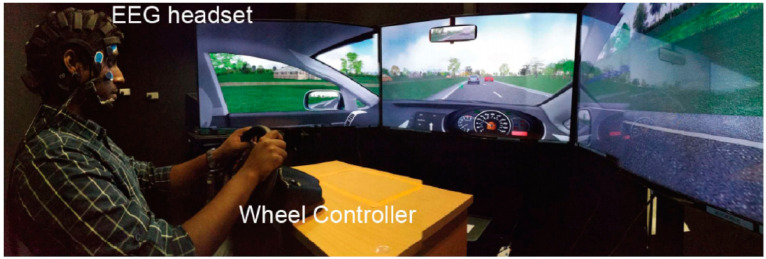
Experiment environment for simulated driving.

In this study, the participants were 20 healthy college students (male/female = 15/5). They were 22.2 ± 3.2 years old. Each participant had never previously taken part in a similar driving fatigue experiment and was right-handed and had a normal or corrected-to-normal vision. All participants should have no mental illness (e.g., epilepsy, schizophrenia, concussion, traumatic brain injury, etc.). In order to make the inside and outside driving environment consistent, all experiments were conducted from 1 p.m. to 5 p.m. Participants were prohibited from consuming caffeine and alcohol 4 h and 24 h before the experiment, respectively. The participants experimented in a quiet environment and simulated real road conditions through the car simulation driving software. Each participant performed a 90-min simulated driving task using a racing wheel (Logitech G27 Racing Wheel, Romanel-sur-Morges, Switzerland), and the simulated driving road environment was displayed on three 65-inch LCD screens, as shown in Figure 1. A guided car was in front of the participants and generated braking signals within two random intervals. Participants need to control the car brake to respond to the signal and maintain a safe distance. The time interval between the brake signal generated by the guide car and the participant’s braking response was recorded as the reaction time (RT). Each participant took part in two identical driving simulation tasks, and 40 segments of experimental data were generated. The study was approved by the Institutional Review Board of the National University of Singapore, and participants signed informed consent before the experiment.

### 2.2. EEG Data Recording and Processing

EEG data were collected using a 24-channel wireless dry headset (Model: HD-72, Cognionics Inc., San Diego, CA, USA) according to the standard 10–20 system. The original EEG signals were collected at 250 HZ, the electrode impedance was controlled below 20 kΩ, and the left and right mastoid was set as the reference electrode. The standard EEG preprocessing procedure was used to process the original signals. Firstly, a band-pass filter (4–40 Hz) was used to eliminate low-frequency DC drifts and 50 Hz power line noise and reset the reference electrode for the original signal using a common average reference. Secondly, fast, independent component analysis (fast ICA) was used to remove the artifacts that are highly correlated with EOG. Finally, the band-pass filter was used to divide the non-artifact signal into four frequency bands: θ (4–8 Hz), α (8–13 Hz), β (13–30 Hz), γ (30–40 Hz). In addition, an individualized window method has been proposed to determine each participant’s state in this study. That is, a five-minute time window with 50% superposition was applied to the RT data, and the time window with the shortest and longest average RT was defined as the wake state and fatigue state [36]. Each five-minute time window has collected 6.41 ± 1.25 (Mean ± SD) RT data. Then the EEG data within these windows were used for subsequent driving fatigue classification, and AFp3h, AFpz, and AFp4h channels were extracted for further processes. For each state, the window/step of 4/2 s was used for EEG section division. Besides, the EEGLAB open-source toolbox [37] was used to preprocess the data on MATLAB 2019b development platform (The MathWorks Inc., Nettiek, MA, USA).

### 2.3. Feature Extracting

The power-spectrum density (PSD), entropy, and functional connectivity (FC) have been widely used as features of EEG driving fatigue detection. In this study, mutual information (MI) was used to estimate the brain FC. Moreover, for each channel of every EEG section, we calculated the four PSD features (α/β, θ/β, (α + θ)/β, (α + θ)/(β + 𝛾)), the approximate entropy (ApEn) of four rhythms, and the MI of four rhythms between every two channels. The detailed calculation formula for these three features is shown in our previous study [36]. Besides, this study innovatively added lateralization features of left and right channel pairs for PSD and ApEn. The lateralization features were evaluated by the asymmetry score [38]: AS(X) = [X(R) − X(L)]/ [X(R) + X(L)], where X(R) and X(L) are the extracted features of the right and left hemispheres, respectively. Three left-right EEG channel pairs, AFp3h- AFp4h, AFp3h-AFpz, and AFpz-AFp4h, were used for PSD and ApEn, resulting in 24 lateralization features. The detailed feature information is shown in Table 1.

### 2.4. Oneformer: Transformer for One-Dimensional Feature Vector

**Figure 2:**
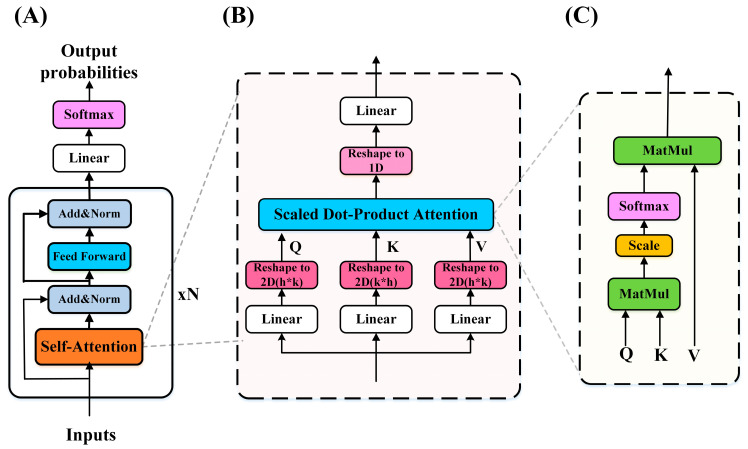
Oneformer architecture. (**A**) Oneformer was composed of N transformer encoders, linear layer, and softmax layer. Each encoder included an improved self-attention sub-block and a standard feed-forward networks (FFN) sub-block. (**B**) Improved self-attention sub-block, which can be used to learn one-dimensional features. (**C**) Standard scaled dot-point attention mechanisms.

The transformer is a unique encoder-decoder structure. Both the encoder and decoder are stacked by multiple transformer blocks. Each Transformer block includes two sub-blocks: multi-head self-attention mechanism and position-wise feed-forward networks. In addition, each sub-block also includes layer normalization modules [39] and residual connectors [40]. Transformer blocks can be used in a different field. Specifically, the encoder-decoder structure can be used for machine translation [32,41], encoder-only can be used in classification problems [42,43], and decoder-only can be applied to the language model [44,45]. In this study, we implemented a transformer variant called Oneformer (Figure 2), which only used the encoder structure to classify a one-dimensional feature vector. Moreover, considering that the feature vector does not contain location information, we did not use positional encoding [32].

#### 2.4.1. Self-Attention Sub-Block for Oneformer

The input of a one-dimensional feature is not suitable for the multi-head attention mechanism (which is the standard component in transformer architecture and is consisted of several parallel self-attention sub-blocks) [32]. Therefore, we chose the self-attention sub-block for Oneformer. Self-attention is able to quantify the interdependent relationship between input features, assigning higher weights to features with strong connections and lower weights to weaker connections. Inspired by the multi-head attention mechanism, we projected the one-dimensional vector into the two-dimensional space so that the model can learn more information (for the scale of the two-dimensional space, we have performed multiple ablation experiments) and used the scaled dot-product attention mechanism for computing. The feature vector X of the previous layer is linearly changed by three groups of weights to obtain the query *XW^Q^*, key *XW^K^*, and value *XW^V^*. The self-attention mechanism is calculated as Equation (1):

(1) $$SelfAttention\left(X\right)=\mathrm{softmax}\left(\frac{XW^{Q}{\left(XW^{K}\right)}^{T}}{\sqrt{k}}\right)XW^{V}$$

where $X\in {\mathbb{R}}^{d_{\mathrm{feature}}}\;\left(d_{\mathrm{feature}}=h\times k\right)$, the *X* of each layer is the output from the previous layer, $W^{Q},W^{K},W^{V}\in {\mathbb{R}}^{d_{\mathrm{feature}}\times h\times k}$ are parameter matrices, $d_{\mathrm{feature}}$ is the features length, h is the width of a one-dimensional feature projected to two-dimensional space, and $\sqrt{k}$ is the scaling factor. 

Then the features of two-dimensional space should be projected into one-dimensional space for subsequent calculation as shown in Equation (2):

(2) $$Attention\left(X\right)=SelfAttention\left(X\right)\times W^{O}$$

where the projections are parameter matrices $W^{O}\in {\mathbb{R}}^{k\times h\times d_{\mathrm{feature}}}$.

#### 2.4.2. Position-Wise Feed-Forward Network Sub-Block for Oneformer

The output of the self-attention module passes through two feed-forward networks and a ReLU activation function. The details are as Equation (3):

(3) FFN(x)=F2(ReLU(F1(x)))

where x is the output of the self-attention module in the form of Wx+b, and F1 and F2 are feed-forward neural networks whose weight parameters are not shared.

#### 2.4.3. Oneformer Block

In each Oneformer block, layer normalization modules and residual connectors were used and expressed as Equations (4) and (5):

(4) $$X_{A}=LayerNorm\left(\mathrm{Attention}\left(X\right)\right)+X$$

(5) $$X_{B}=LayerNorm\left(\mathrm{FFN}\left(X_{A}\right)\right)+X_{A}$$

where X and $X_{B}$ are the input and output of each Oneformer block.

### 2.5. Gating Mechanism in Oneformer

Recently, several studies have proposed improvement measures to improve the performance of the transformer, and GLU has been adopted to obtain good performance [46,47]. GLU is used to pass the input X through two linear projections, one of which passes through the sigmoid function and finally multiplies the outputs of the two components [48]. GLU can recalibrate the previously learned features, adaptively activate the features, and select the features with strong discriminants. In this study, we added GLU in the self-attention sub-block and FFN sub-block, called GLU-Attention and GLU-FFN. Here we used GLU without bias version [49] shown in Equation (6):

(6) $$\mathrm{GLU}\left(X\right)=\phi \left(XW_{u}\right)\;⊗\;XW_{v}$$

where ϕ denotes the GELU activation function, ⊗ refers to the point-wise multiplication, $W_{u},W_{v}\in {\mathbb{R}}^{d_{\mathrm{feature}}\times d_{\mathrm{feature}}}$. 

#### 2.5.1. GLU-FFN in Oneformer

**Figure 3:**
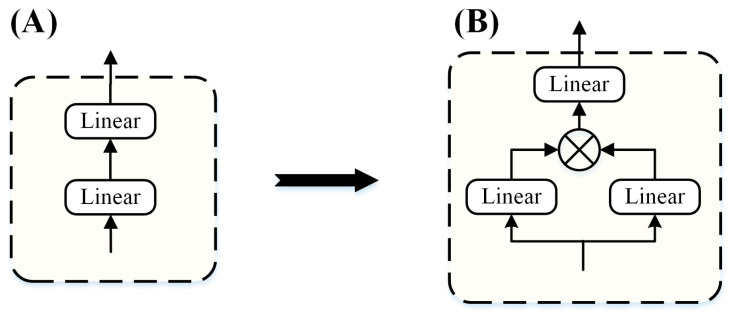
GLU-FFN. (**A**) FFN module of the Oneformer. (**B**) GLU was introduced into the FFN sub-block of the Oneformer.

For the FFN sub-block, we adopted the improved method proposed by Shazeer to replace the first linear layer and activation function [49], as shown in Figure 3. The activation function used Gaussian Error Linear Unit (GELU), which allows partial negative weights to pass, while ReLU suppresses all negative weights. Among these negative weights, some information may be more important for subsequent learning. GLU-FFN is calculated as Equation (7):

(7) $${\mathrm{FFN}}_{\mathrm{GLU}}\left(X\right)=\mathrm{GLU}\left(X\right)W_{t}$$

where $W_{t}\in {\mathbb{R}}^{d_{\mathrm{feature}}\times d_{\mathrm{feature}}}$.

#### 2.5.2. GLU-Attention in Oneformer

**Figure 4:**
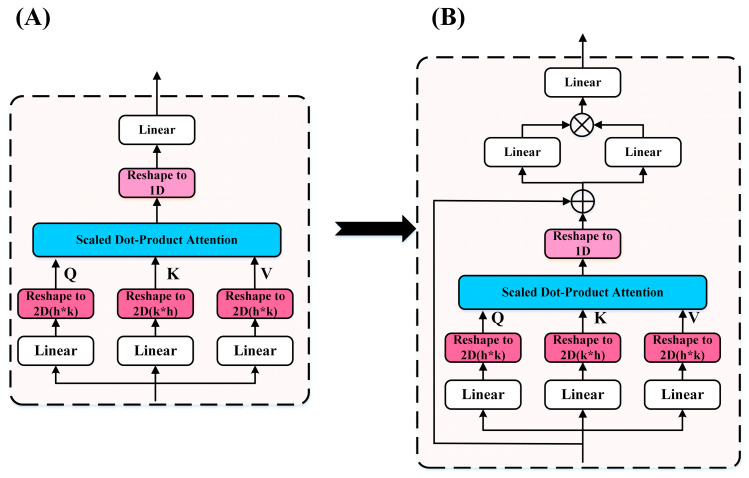
GLU-Attention. (**A**) Self-attention module of the Oneformer. (**B**) GLU was introduced into the Attention sub-block of the Oneformer.

We also applied GLU to the Attention sub-block, as shown in Figure 4. Here, we used the residual connection [40] to fuse the context information through the self-attention module, which can improve the stability of the model. The fused information is passed through GLU to improve the performance of the Attention sub-block. GLU-Attention is calculated as Equation (8):

(8) $$Attention_{GLU}\left(X\right)=\;\mathrm{GLU}\left(SelfAttention\left(X\right)+X\right)$$

### 2.6. GLU-Oneformer Parameters

For the Oneformer model, the batch size was set as 2048, and the model was trained for 1000 epochs with early stopping. Layer normalization and dropout were added to the output of each sub-block in the Oneformer, and $P_{\mathrm{drop}}$ = 0.3. In addition, the AdamW optimizer was used for gradient optimization with β1 = 0.9, β2 = 0.98, and the weight decay was 0.01. 

**Figure 5:**
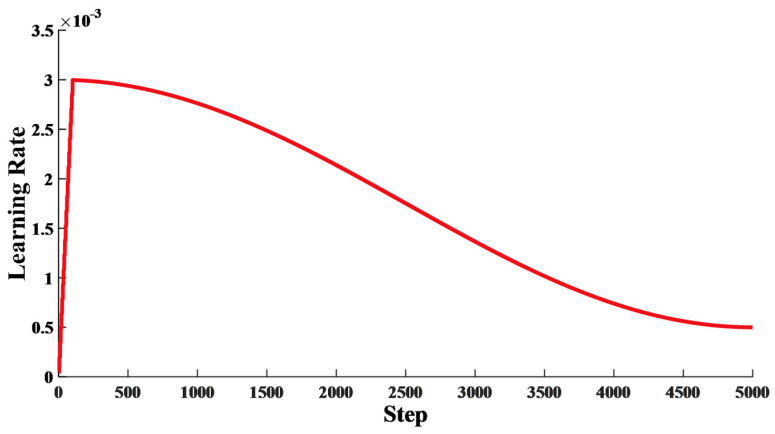
Learning rate setting during model training with warming strategy. According to the set batch size, our training samples were trained five steps per epoch, and a total of 5000 steps were trained in 1000 epochs.

For the learning rate of Oneformer, we adopted the warmup strategy shown in Figure 5. The initial learning rate was set to 5 × 10^−5^, gradually increased to 3 × 10^−3^ after 20 warmup epochs, and finally gradually decreased to 5 × 10^−4^. At the beginning of training, the weight of the model is randomly initialized, and a low learning rate can improve the stability of the model. At the end of the warmup, a large learning rate can accelerate the convergence of the model and improve performance. At the later stage of training, the performance of the model is stable, and a large learning rate will cause shock.

### 2.7. Oneformer Architecture Optimization

Setting different parameters for each component in the Oneformer architecture will affect the model identification performance. The artificial selection method and Tree-of-Parzen-Estimators (TPE) hyperparameter search method was used to optimize the parameters of the improved Oneformer architecture and find the optimal Oneformer variant. For the artificial selection method, we conducted ablation experiments on the basis of the base model (size_h = 6, num_encoder = 6, and num_hidden = 256), which was the method used by Vaswani et al. to find the optimal Transformer variant [32]. For comparison, the TPE method, which is a hyperparameter search method based on Bayesian optimization [50] in hyperopt python frame [51], was proposed to automatically search for the optimal parameter combination of Oneformer. There are three steps for automatically optimizing the hyperparameters. Firstly, define the objective function to be optimized. The final classification accuracy of Oneformer was taken as our optimization goal. Secondly, define a configuration space. The TPE method supports continuous, discrete, and conditional variable optimizations. In this study, the variables and optimized ranges of the Oneformer are shown in Table 2. Thirdly, set the search number. The maximum search number was set to 60.

### 2.8. Machine Learning Classifiers

The support vector machine (SVM) [52], K-NearestNeighbor (KNN) [53], and random forest (RF) [54] were used to analyze and verify the influence of lateralization on the accuracy of driving fatigue recognition. (1) SVM is to build a decision function to classify data. For nonlinear data, SVM projects the data into a high-dimensional linear feature space by a kernel function and constructs an optimal classification hyperplane in this high-dimensional space to obtain the decision function of the classifier. (2) KNN is to calculate the distance between the test sample and the training sample and select the K training samples closest to the test sample and determine the category with the largest number of these K samples as the category of the test sample. (3) RF is an ensemble algorithm based on the decision tree, which extracts m subsets from the training set by bootstrapping strategy and uses m decision trees to train them. For the classification task, the voting strategy takes the category with the most votes as the output value. In this study, SVM adopted the RBF kernel function, the number of nearest neighbors of KNN was set to 3, and the number of decision trees in RF was set to 500.

### 2.9. Deep Learning Classifiers

Some typical deep learning algorithms are used in comparison with our model for one-dimensional feature vector classification, including one-dimensional CNN (1D CNN) [55], LSTM [56], Bidirectional LSTM(BiLSTM) [57], Gate Recurrent Unit (GRU) [58], Bidirectional GRU (BiGRU) [59]. Brief descriptions for each baseline are as follows. (1) 1D CNN uses a unique convolution structure to capture the nonlinear relationship between local features and has good local information processing ability. (2) LSTM is a variant of recurrent neural networks (RNN), which solves the problem of long-term dependence on signals. It can store and record time information in sequence information. (3) BiLSTM model is a variant of the LSTM model, which is composed of a forward LSTM model and a backward LSTM model. It can obtain past information ignored by the LSTM model. (4) GRU model is a variant of the LSTM model that has a simpler structure and fewer parameters than LSTM, which has less training time and low over-fitting probability. (5) Similarly, BiGRU consists of a forward GRU model and a backward GRU model. 

**Figure 6:**
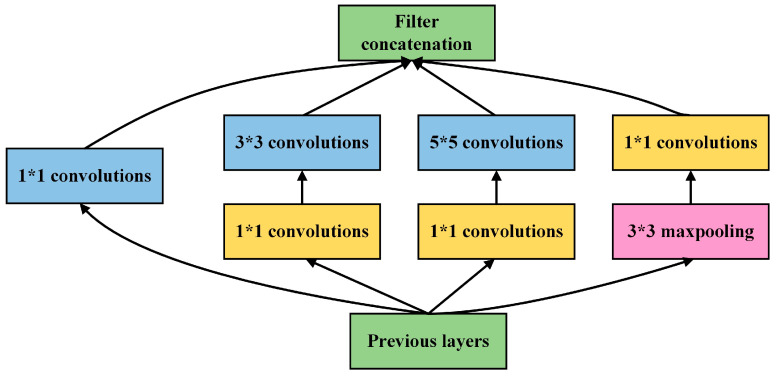
The basic inception structure.

**Figure 7:**
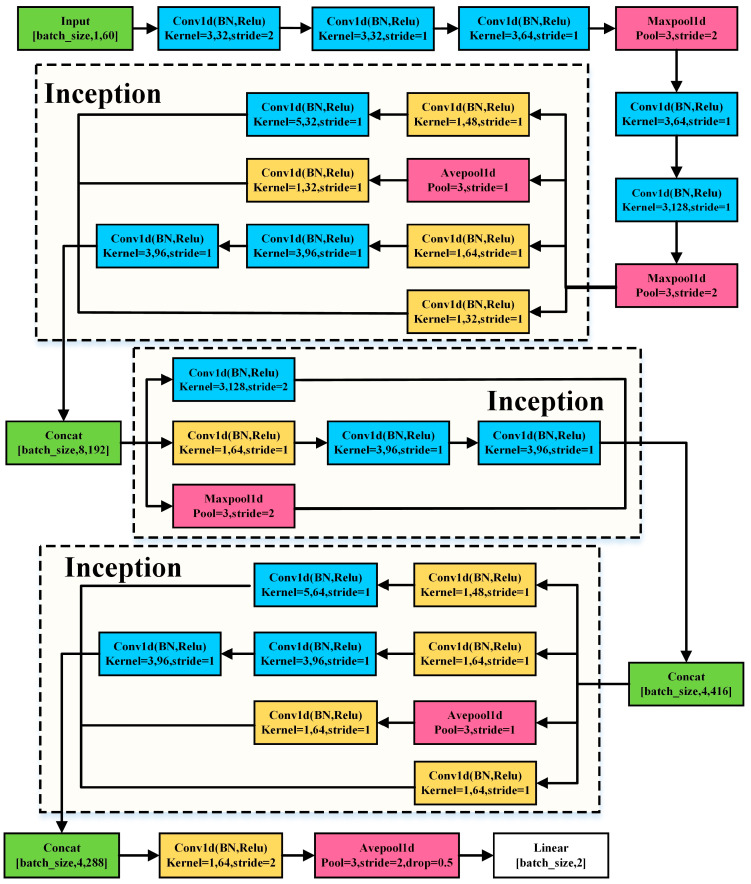
The 1D Inception structure. BN represents batch normalization.

In addition, we propose an improved Inception structure that extracts features at different scales by multiple convolution kernels of different sizes for one-dimensional feature vector classification, called 1D Inception. Previous CNNs have increased the depth of the model to improve its performance of the model. However, the Inception structure [60] not only increases the depth of the model but also increases the width of the model to improve its performance. In Inception, the large convolution kernel is replaced with several parallel small convolution kernels for operation. The basic Inception structure is shown in Figure 6. While increasing the operation speed, it can connect different outputs together and adaptively select the required information through the weights of the next layer network. In addition, the Inception structure uses a 1 × 1 convolution kernel to reduce the parameters of the model and improve model performance. In this study, our 1D Inception (shown in Figure 7) used three different Inceptions to learn the features, and all convolution kernels used one-dimensional convolution. Moreover, all padding in convolution and pooling structures selects the same type. 

Besides, the hyperparameters (including learning rate, batch size, epochs, optimizer, and weight decay) of these models were set the same as in the GLU-Oneformer. Similarly, the TEP method was used to search the structure of the temporal neural network to find the optimal architecture. Table 3 shows the parameters and ranges searched by the TPE method.

### 2.10. Evaluation Metrics for Classifiers

Repeated 5-fold cross-validation was used to reduce the bias of the classification results. Four folds of the data were used for training, and one-fold was used to test the model to evaluate its performance of the model. Ten repetitions were conducted for averaging to obtain the final evaluation results. Accuracy, precision, recall, and f1-scores were calculated to evaluate the performance of the model. The true positives (*TP*), false negatives (*FN*), true negatives (*TN*), and false positives (*FP*) were obtained by comparing prediction labels with real labels. Specifically, *TP* indicates the number of positive labels predicted as positive labels, *FN* indicates the number of positive labels predicted as negative labels, *FP* indicates the number of negative labels predicted as positive labels, and *FT* indicates the number of negative labels predicted as negative labels. These four model evaluation indexes are calculated as Equations (9)–(12):

(9) $$Accuracy=\frac{TP+TN}{TP+TN+FP+FN}$$

(10) $$Precision=\frac{TP}{TP+FP}$$

(11) $$Recall=\frac{TP}{TP+FN}$$

(12) $$F_{1}=\frac{2TP}{2TP+FP+FN}$$

## 3. Results and Discussion

In this study, we combined the lateralization features and the improved transformer architecture of GLU-Oneformer to support NHB montage to achieve high accuracy for fatigue driving detection. The main findings are as follows. Firstly, we used the personalized scheme to extract the wake and fatigue states to reduce the influence of individual differences and confirmed the generation of driving fatigue by the significant change in RT. Secondly, the innovative application of brain lateralization to extract lateralization features for fatigue detection can significantly improve the performance of classification. Thirdly, we proposed a GLU-Oneformer architecture, which can well capture the global information of a one-dimensional feature vector for classification, to further improve the performance of the classification model. The results are shown and discussed in greater detail below.

### 3.1. Driving Fatigue Determination with Behavioral Performance

**Figure 8:**
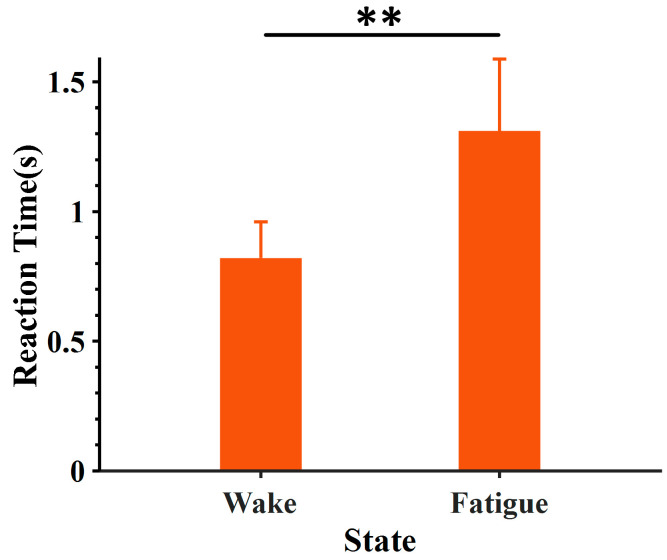
The mean RTs corresponding to wake and fatigue states were extracted by the individualized window method. The error bar represents the standard deviation of all participants, and ** represents *p* < 0.01.

In this study, we used a personalized approach to determine the wake and fatigue states based on individual RT. It has been reported that behavioral performances can reflect the fatigue state greatly [61]. The level of fatigue will continue to accumulate as the simulated driving task progresses, and it will also show a continuous increase in RT. Generally, most researchers choose the beginning and end of the experiment as the wake and fatigue states [8]. In fact, our previous research has shown that there were different trends in the development of fatigue degrees among participants [36]. Therefore, we defined the minimum and maximum RT as the personal wake and fatigue states at the individual level. This personalized approach can reduce the impact of individual differences. As shown in Figure 8, there were significant differences in RT between the defined wake and fatigue states through the one-way analysis of variance (RT_wake_ < RT_fatigue_, F = 100.97, *p* < 0.01). Behavioral results demonstrated that the designed experiment successfully induced driving fatigue, and EEG data of the selected time window can be used for further classification.

### 3.2. Improvement with Lateralization Feature

The classification performance of adding lateralized features is shown in Table 4. Our previous research used the features of the three NHB EEG channels (excluding lateralization features) and obtained the highest classification accuracy of 76.47% with the RF model [36]. After adding lateralization features, the driving fatigue recognition accuracy ascended to 82.10% with the SVM model, along with the recall of 82.03%, precision of 82.22%, and F1-score of 82.04%. In addition, a one-way analysis of variance revealed significant differences before and after the addition of the lateralization feature (F = 456.56, *p* < 0.01), indicating the effectiveness of the lateralization feature on model performance improvement. The extraction of lateralization features was based on brain lateralization theory. Related studies have reported the right-lateralized activation of sustained attention tasks in the frontal control area [62,63], which is the direct result of top-down signal transduction [11]. This suggests that the left and right brains have different contributions during the process of the simulated driving task. The effectiveness of the lateralization feature can help the classifiers learn this difference and improve the performance of the models for driving fatigue detection.

### 3.3. Classification with Oneformer

Table 5 shows the optimal Oneformer variants of the artificial selection method and the TPE method. Compared with the artificial selection method (Accuracy:85.65%, Precision:83.78%, Recall:83.77%, and F1-score:83.77%), the TPE method achieves the highest model performance (Accuracy: 85.92%, Precision: 84.11%, Recall: 84.12%, and F1-score: 83.88%). In addition, since the target parameters are generally non-convex, the artificial selection method often requires more time and computing resources to try various parameter combinations, which is easy to fall into the optimal local solution [64,65]. TPE method can realize the fast optimization of hyper-parameters and can effectively avoid the optimization results falling into the optimal local solution, which gives the prediction model good generalization ability [66]. Chai et al. proposed fuzzy particle swarm optimization with cross-mutated (FPSOCM) to optimize artificial neural networks, significantly improving recognition accuracy of mental fatigue classification compared to their previous studies [67].

Moreover, the ablation experiments of Oneformer on the basic model were implemented to explore the effects of different component parameters on the model performance shown in Table 5. As shown in Table 5 rows (A), we explored the dimension of projecting a one-dimensional vector into two-dimensional space. The parameters of *h* and *k* are the width and length of two-dimensional space, simulating the number of heads and the dimension of each head in the original transformer. When *h* = 1, it means that only a one-dimensional feature vector is used to calculate attention, and the performance of the model is the lowest, which proves the reliability of our feature projection into two-dimensional space. The performance of the model will not improve with the increase in *h*, and only the performance of the model with the appropriate two-dimensional size is optimal. In Table 5, rows (B), we explore the role of the number of hidden layers in the fully connected layer. When the number of hidden layers increases to 256, the performance of the model is the best. In Table 5, rows (C), we explore the influence of the number of coding layers on the performance of the model. Increasing the number of encoders will increase the performance of the model, but the complexity of the model and the computation time will also increase. Meanwhile, in the variants of multiple Oneformers, Precision, Recall, and F1-score are relatively equal, indicating that the performance of the model is relatively stable and does not tend to a certain category.

**Figure 9:**
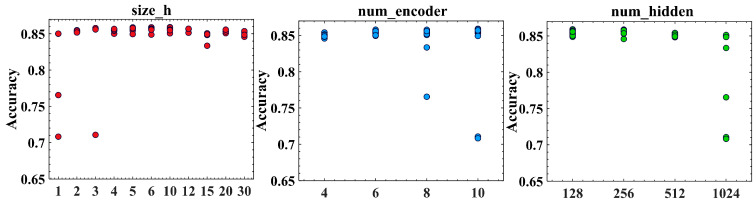
Performance of parameter combination optimized by TPE method.

In this study, we took accuracy as the optimization objective of the model, and the trial function in the hyperopt frame was used to record more details using for analyzing the metrics in the process of program optimization [51]. The details of the TPE optimization are shown in Figure 9. When a one-dimensional vector is used to calculate attention, the performance of the model will decrease significantly, which is consistent with the previous ablation experiment results. When using two-dimensional size, different parameter combinations between size_h, num_encoder, and num_hidden have little effect on the performance of the model. With the number of encoders increasing, the performance of the model will be slightly improved. In addition, with the number of hidden layers of the FFN layer increasing, the performance of the model will be slightly decreased. When size_h = 10, num_encoder = 10, and num_hidden = 128, the model obtains the highest performance. 

### 3.4. Driving Fatigue Detection with GLU-Oneformer

Table 6 shows the performances of the deep learning models and the improved Oneformer after adding the GLU. For deep learning models, our proposed 1D Inception achieves a higher classification performance with an accuracy of 76.83%, a precision of 75.57%, a recall of 75.73%, and an F1-score of 75.37% compared to typical CNN and RNN variants. However, the performances of these deep learning models were lower than that of traditional machine learning models (Accuracy: 82.10%, Precision: 82.22%, Recall: 82.03%, and F1-score: 82.04%). Although the deep learning algorithm performs well in images and speech, it is still a challenge in the classification of one-dimensional feature vectors [68,69]. Shwartz-Ziv et al. pointed out that deep neural networks are not suitable for all types of one-dimensional feature vector data, and the probable reason is that the inductive bias of deep learning does not apply to modeling this type of data [70,71]. The unique convolutional structure of CNN is more suitable for processing data with translation invariance, such as pictures. However, for one-dimensional feature vectors, different feature placement orders represent the same meaning, but the information learned by CNN is completely different. In addition, the unique memory unit of LSTM is more suitable for processing time series data, but the one-dimensional feature vector does not contain time information. Moreover, in one-dimensional feature vectors, there are usually irregular and complex correlations between features [72], and the rotational invariance of deep learning is difficult to learn such data [73].

The unique self-attention mechanism of the Oneformer can better deal with the global information of one-dimensional feature vectors, capture the relationship between features greatly, and do not need the data to be translation invariant. In particular, projecting one-dimensional features into high-dimensional space and modeling the interdependencies among the features can teach richer spatial information, which significantly improves the quality of extracted features. Here, GLU was introduced into the Onerformer of self-attention sub-block and FFN sub-block (called as GLU-Oneformer) to recalibrate the learned features and activate features with high resolution, expecting to improve the performance of the Oneformer model. For the variants in GLU-Oneformer, we also use the validated TPE method to optimize the model parameters. As shown in Table 6, we can see that both GLU-Attention and GLU-FFN can improve the Oneformer performance after adding the GLU, achieving the accuracies of 86.43% and 86.57%, respectively, which are higher than the Oneformer alone (85.92%). Furthermore, GLU-Oneformer was proven to obtain the highest classification performance. This simple attention mechanism can improve the model performance without increasing too much computation compared with the basic transformer structure.

Although many previous studies have achieved high driving fatigue recognition performance using EEG data from the whole brain channel [24,25], it is still difficult to apply it to actual driving scenarios. Studies have shown that EEG signals in the forehead channel are more sensitive to changes in the driver’s fatigue state [74,75], which is a potential area to effectively determine when the driver, which is a potential area to effectively determine when the driver is fatigued. Therefore, only using fatigue-related forehead leads [67] or NHB strategies [15] can effectively improve the practicality of driving fatigue detection, which will promote the development of wearable devices for fatigue warnings. Mu et al. fused the fuzzy entropy of FP1 and FP2 forehead electrodes and obtained 85% recognition performance [76]. Qin Wei et al. obtained 80.0% classification accuracy by using the NHB strategy combined with the SVM algorithm [15]. In our previous study, we proposed a channel pair sorting method to theoretically verify the effectiveness of the NHB strategy, but the classification performance is only 76.47% [36]. In this study, we further proposed a GLU-Oneformer classification algorithm and combined it with the introduced brain lateralization feature to achieve an accuracy of 86.97%, which significantly improved the recognition performance of fatigue state and can support the NHB strategy for practical application greatly.

### 3.5. Limitations

In this study, the current fatigue driving detection methods based on NHB montage and GLU-Oneformer architecture have achieved great recognition performance, but some limitations still need to be considered. Firstly, the EEG data were obtained by a simulated driving experiment. Although the experimental results demonstrated that the fatigue state was successfully induced and the effectiveness of the experimental scheme was verified, it cannot be compared with the complex driving environment of the actual road. Secondly, 40 segments of experimental data were obtained. For the classification models, the collected samples are not large enough, and the robustness of GLU-Oneformer is difficult to verify effectively. Thirdly, we only use the time window of 4 s to calculate the EEG features without fully exploring the impact of other time window divisions on the classification results. In subsequent studies, we will continue to try other time window divisions (2s, 6s, 8s, etc.) to discuss the detection performance of the model. In summary, it is still far from practical application at present. We need to collect more actual road data to further verify our method in the future. 

## 4. Conclusions

In this study, practicability and accuracy have been concerns for driving fatigue detection. NHB montage with AFp3h, AFpz, and AFp4h EEG channels with high practicability has been theoretically proved to be valid by our previous study. To further improve the accuracy of driver fatigue recognition, we introduced the lateralization features on the basis of our previous feature set and proposed the GLU-Oneformer architecture. The main conclusions of this study are as follows. Firstly, we have proved that the lateralization features can significantly improve the classification performance, which improved the recognition accuracy of the model by 5.63% and achieved an accuracy of 82.10%. Secondly, the TPE method can gain the best Oneformer framework compared with the artificial selection method and achieve an accuracy of 85.92%. Thirdly, our proposed GLU-Oneformer achieved better performance compared with typical deep learning models (1D CNN variants and RNN variants) and common machine learning models on the basis of the global information extraction ability. Fourthly, GLU-Oneformer obtained the highest performance of 86.97% for driving fatigue detection, which improved the recognition accuracy by 1.05% compared with the Oneformer model, indicating that GLU could improve the performances of the self-attention sub-block and FFN sub-block. In our future work, we hope to develop a practical system based on our method to reduce the occurrence of traffic accidents.

## Figures and Tables

**Table 1:** The EEG features set was used in this study.

	PSD	Entropy	FC	Lateralization
Features	α/β, θ/β, (α + θ)/β, (α + θ)/(β + γ)	ApEn	MI	[X(R) − X(L)]/ [X(R) + X(L)]
Numbers	4 × 3	4 × 3	4 × 3	(4 + 4) × 3

Note: We used three EEG channels and four frequency bands. The PSD and ApEn were calculated using a single channel, and MI was computed between two EEG channels. So the PSD and ApEn of the left and right channel pairs were used to calculate the lateralization features.

**Table 2:** The variables and optimized ranges of TPE method.

Variables	Choice
size_h	[1,2,3,4,5,10,12,15,20,30]
num_encoder	[4,6,8,10]
num_hidden	[128,256,512,1024]

**Table 3:** The variables and optimized ranges of the TPE method for the temporal neural network.

Variables	Choice
num_layers	[1,2,3,4]
num_hidden	[50,100,150,200]

**Table 4:** Classification performance of adding lateralization feature with machine learning models.

Models	Accuracy (%) (Mean ± SD)	Precision (%) (Mean ± SD)	Recall (%) (Mean ± SD)	F1 (%) (Mean ± SD)
KNN	79.47 ± 0.19	81.56 ± 0.21	78.28 ± 0.20	79.88 ± 0.20
SVM	**82.10 ± 0.23**	**82.22 ± 0.25**	**82.03 ± 0.24**	**82.04 ± 0.25**
RF	76.70 ± 0.23	76.74 ± 0.24	76.63 ± 0.26	76.74 ± 0.24

**Table 5:** Variations on the Oneformer architecture.

	*N*	*d_ffn_*	*h*	*k*	Accuracy	Precision	Recall	F1-Score
Base	6	256	6	10	85.42%	83.64%	83.27%	83.45%
(A)			1	60	85.02%	83.08%	83.23%	83.10%
			2	30	85.38%	83.51%	83.22%	83.42%
			3	20	85.36%	83.43%	83.27%	83.36%
			4	15	85.35%	83.38%	83.30%	83.33%
			5	12	85.41%	83.49%	83.17%	83.32%
			10	6	85.38%	83.59%	83.06%	83.28%
			12	5	85.41%	83.65%	83.14%	83.38%
			15	4	85.33%	83.31%	83.23%	83.41%
			20	3	85.27%	83.36%	83.03%	83.28%
			30	2	85.19%	83.43%	82.92%	83.17%
(B)		128			85.38%	83.58%	83.30%	83.43%
		512			85.40%	83.62%	83.16%	83.43%
		1024			85.07%	83.16%	82.87%	83.00%
(C)	4				85.24%	83.28%	83.45%	83.35%
	8				85.46%	83.67%	83.64%	83.49%
	10				85.62%	83.72%	83.73%	83.71%
Best	10	512	6	10	85.65%	83.78%	83.77%	83.77%
TPE	10	128	10	6	**85.92%**	**84.11%**	**84.12%**	**83.88%**

**Note:** Values not listed are the same as those of the base model.

**Table 6:** The performances of the deep learning models and the improved Oneformer after adding the GLU.

Model	Accuracy (%) (Mean ± SD)	Precision (%) (Mean ± SD)	Recall (%) (Mean ± SD)	F1 (%) (Mean ± SD)
1D CNN [55]	73.86 ± 0.97	72.93 ± 1.19	74.10 ± 1.31	73.56 ± 0.75
LSTM [56]	71.74 ± 1.15	71.28 ± 1.34	70.83 ± 1.36	69.81 ± 1.12
BiLSTM [57]	72.19 ± 1.03	71.62 ± 1.11	71.68 ± 1.22	71.73 ± 0.93
GRU [58]	73.02 ± 1.28	72.89 ± 1.27	72.31 ± 1.29	72.43 ± 1.07
BiGRU [59]	73.74 ± 1.12	73.53 ± 1.18	73.81 ± 1.38	73.92 ± 0.99
1D Inception	76.83 ± 1.08	75.57 ± 1.23	75.73 ± 1.25	75.37 ± 0.94
GLU-Attention	86.43 ± 0.59	84.61 ± 1.06	84.22 ± 1.53	84.32 ± 0.79
GLU-FFN	86.57 ± 0.59	84.75 ± 1.03	84.39 ± 1.27	84.56 ± 0.75
GLU-Oneformer	**86.97 ± 0.43**	**85.08 ± 0.09**	**85.44 ± 1.13**	**85.23 ± 0.67**

**Note:** The GLU-Attention and the GLU-FFN are models after adding GLU to the self-attention sub-block and FFN sub-block in Oneformer, and GLU-Oneformer is a model after applying GLU to both sub-blocks in Oneformer.

## Data Availability

Not applicable.
